# Emphasizing the role of cardiothoracic surgeons within the global health context: a call to action

**DOI:** 10.1097/MS9.0000000000003678

**Published:** 2025-08-05

**Authors:** Achanga Bill Smith Anyinkeng, Abdullah K. Alassiri, Jonas L. Ibekwe, Efuetlateh John Paul Nchonganyi, Samuel G. Junior Fodop, Effiom Victory Bassey, Kelechi E. Okonta

**Affiliations:** a Faculty of Health Sciences, University of Buea, Buea, Cameroon; b Association of Future African Cardiothoracic Surgeons (AFAC), Yaounde, Cameroon; c Faculty of Medicine, King Abdulaziz University, Jeddah, Saudi Arabia; d Department of Clinical Medicine and Surgery, Faculty of Clinical Sciences, College of Medicine, University of Ibadan, Ibadan, Nigeria; e Department of Surgery, Mbingo Baptist Hospital, Bamenda, Cameroon; f Catholic University of Cameroon, Bamenda, Cameroon; g Faculty of Clinical Sciences, University of Calabar, Calabar, Nigeria; h Division of Cardiothoracic surgery, University of Portharcourt Teaching Hospital, Portharcount, Nigeria

**Keywords:** action, cardiovascular, global health, low- and middle-income country, policymaking

## Abstract

**Introduction:**

Worldwide, cardiovascular thoracic diseases (ischemic heart diseases) are the second most common global cause of disability-adjusted life years (DALYs. Globally, 17 million lives are lost annually, and 80% occur in LMICs as a result of CVD, and 75% of the world lacks access to cardiac surgery with some regions (Sub-Saharan Africa) having 0.07 pediatric cardiac surgeons and 0.12 adult cardiac surgeons per million population. This is a global cause for concern and needs immediate attention.

**Method:**

Through literature review, we elaborate on key points such as the global burden of cardiothoracic surgical diseases, the current status of cardiothoracic surgical care around the world, and the inherent benefits of building a strong cardiothoracic surgical care capability for health systems globally. Search engines used were: PUBMED, SCOPUS, MEDLINE, Cochrane, and Google Scholar.

**Results:**

Cardiothoracic diseases make up an important part of the global health burden of diseases as cardiothoracic care can be linked to over 15 of the 17 Sustainable Development Goals, giving the tremendous part played by cardiothoracic surgeons in the Health System Surgical Initiatives.

**Conclusion:**

We, therefore, recommend policymakers and global health actors: increase the involvement of cardiothoracic surgeons within the global health systems and dialogues; integrate and enforce cardiothoracic surgical care within the global surgery movement; and most importantly promote the training and education of cardiothoracic surgeons in LMICs in the field of global public health and as well involve cardiothoracic surgeons in the national surgical system strengthening process.

## Introduction

Cardiovascular diseases (CVD) are responsible for 17 million deaths annually across the world, more than two-thirds of which are in low and middle-income countries (LMICs). This uncontrolled rise in CVD burden puts an additional burden on the already inadequate cardiac health care system and socioeconomic resources^[1]^. While the need for cardiac surgery shows a falling trend in high-income countries (HICs), the need is alarmingly high in LMICs housing almost 6.4 billion people while cardiac surgical facilities and the number of cardiac surgeons address only 2% and 1% of the population needs respectively^[2]^. This coupling of high CVD burden with inadequate cardiothoracic surgical care due to economic strain and lower surgical expertise in LMICs emphasizes the need for global cooperation for the benefit of highly affected regions.

**HIGHLIGHTS**

- Cardiovascular diseases (CVD) are responsible for 17 million deaths annually across the world, more than two-thirds of which are in low and middle-income countries.
- Cardiothoracic surgeon’s involvement in the national health policy is crucial in improving access to cardiothoracic surgery within low-income countries.
- The unfavorable growth index in most low-income countries, impedes the establishment of new and maintain the infrastructure used in cardiothoracic surgery.
- Advocacy, policymaking, and funds should be allocated for such surgeries.

Currently, global cooperation centers for cardiac surgeries are operating in several countries in the accomplishment of WHO goals for sustainable development and health for all through global coordination. Lee Centre in South Korea provides grounds for shared knowledge between HIC and LMIC health teams, visiting teams to LICs (Low-Income Countries) to share surgical burden as well as in-hospital training programs in South Korean or LMIC facilities^[3]^. Pan-African Society for Cardiothoracic Surgery (PASCaTS) is a similar platform that provides distant and in-site training and mentorship to LICs cardiac surgeons^[4]^. Recent developments include: the Cardiac Surgery Intersociety Alliance (CSIA), a collaborative establishment of cardiac surgical societies, including the American Association for Thoracic Surgery (AATS), Asian Society for Cardiovascular & Thoracic Surgery (ASCVTS), European Association for Cardio-Thoracic Surgery (EACTS), Society of Thoracic Surgeons (STS), and the World Heart Federation which is functionally operational in supporting LMIC cardiac surgery centers, Global Cardiac Surgery Initiative (GCSI) which is a global social cooperation platform bringing both cardiac surgery trainers and trainees under its net, the National Institutes of Health’s “National Heart, Lung, and Blood Institute” (NHLBI) which has been a source of training as well as financial aid to African countries for Rheumatic heart disease (RHD) operations and Thoracic Surgery Foundation (TSF) which provides funded fellowships and conferences to cardiac surgery trainees across the world^[5]^.

Despite all these efforts, cardiac surgical care services are still unable to meet population needs or cater to disease burden in LMICs, particularly after its negligence in the World Health Assembly’s agenda precipitated by the lack of globalization of the cardiac health community and insufficient political and administrative support^[6,7]^. This review in line with the TITAN checklist^[8]^, did not use any artificial intelligence in the write-up of this work, and the work aims to explore the global burden of cardiac surgery, assess existing surgical capacity and highlight the necessity of international collaboration. Additionally, it examines the benefits of a globally integrated cardiac surgical community while addressing potential challenges to its realization.

## Methods

This study is a narrative review. Through a comprehensive literature review, we utilized multiple search engines, such as PUBMED, MEDLINE, SCOPUS, Cochrane, and Google Scholar. We extensively searched existing literature using search terms such as: “global burden of cardiothoracic surgical disease,” “current status of cardiothoracic surgical care,” and “inherent benefits of cardiothoracic surgical care” to health systems. Our search terms were optimized using Boolean operators and MESH terms. The citations were downloaded and exported directly to Zotero to screen articles for eligibility.

Inclusion criteria: Open-access English papers published in peer-reviewed journals and official cardiothoracic websites that match our search terms.

Exclusion criteria: Commentaries and opinion papers published in peer-reviewed journals. As well as non-peer-reviewed articles.

## Results

### Global burden of cardiothoracic surgery diseases and the disproportional effect on lMICs

Cardiovascular diseases are one of the leading causes of morbidity and mortality globally claiming almost 18 million lives along with the loss of 366 million disability-adjusted life years (DALYs) every year while 80% burden of disease lies in LMICs^[9]^. In 2021 alone, ischemic heart disease caused 9.5 million deaths worldwide becoming the most prevalent cardiovascular disease^[10]^.

Rheumatic heart disease (RHD) is another alarmingly prevalent health disease affecting 30 million patients worldwide with a mortality of 0.4 million patients per year^[3]^. The last two decades have seen an almost 14% rise in RHD incidence and prevalence in LMICs but a downward trend in HICs^[11]^. The continual burden of valvular repair surgeries in RHD-affected areas is a challenge particularly in LMICs due to insufficient infrastructure and fewer experts^[3]^.

Despite the heavy burden and high mortality, there is inadequate availability of cardiothoracic surgical services, especially in LMICs. A study conducted in 2019 reported 34.8, 149.0, and 271.9 centers per million surgical CVD incidence in low-income, lower-middle-income, and upper-middle-income countries, which is quite low as per population and disease burden^[12]^. Globally, 1.64 and 0.52 cardiac surgeons exist per million adult and pediatric populations respectively while the number drops to 0.04 per million adult population in low-income countries^[13]^.

A comprehensive review of the UN’s sustainable development goals (SDG) has reported that 15 out of 17 SDG goals are notably affected by cardiosurgical care and community due to high CVD burden while cardiac surgery facilities do not meet 93% of the population’s needs residing mainly in LMICs^[14]^. All these reports emphasize the need for global collaboration to avert disease burden, particularly in LICs where economic and political conditions do not allow accessibility of high-volume cases to surgical facilities^[6]^.

### The current state of cardiothoracic care around the world: workforce deficit and lack of cardiothoracic building in current global health efforts

The field of cardiothoracic surgery has undergone remarkable changes in the last decade, from an increase in the number of specialists to the modernization of cardiothoracic surgical interventions around the globe. However, to date, cardiothoracic surgery still records an overwhelming unmet need. LMICs have the greatest unmet cardiothoracic surgical disease burden, attributed to the small ratio of cardiothoracic surgeons per patient population, and the difficulty in accessing cardiothoracic surgical care. There are quite some bottlenecks that contribute to poor access to cardiothoracic surgical services, some of which include insufficient infrastructure, inadequate training, limited workforce, the geographical location of these centers, and political unrest in some of these countries^[15,16]^.

Although cardiovascular diseases are the leading cause of morbidity and mortality, with an estimated one-third of this burden requiring surgical or interventional care, the cardiothoracic surgical workforce is yet to meet the unmet burden of these diseases^[17,18]^. Despite the increasingly growing workforce density seen in many countries around the globe, LMICs still have the lowest cardiothoracic surgical workforce density globally^[17]^. With 62.7% of the active workforce of cardiothoracic surgeons aged >55 years and nearing retirement^[19]^, there is now substantial concern about the future supply of cardiothoracic surgeons to meet the burden of cardiovascular diseases whose numbers will double between now and 2030^[20]^. An analysis of the number of cardiac surgeons, conducted in 2022, found that HICs have approximately 7.15 cardiac surgeons per million people compared to 0.04 in LMICs. Similarly, there are 9.51 pediatric cardiac surgeons per million pediatric population in HICs compared to 0.07 in LMICs^[18]^. This mal-distribution was not different for cardiothoracic surgeons, with 27 and 28 cardiothoracic surgeons per million population in North America and Western Europe, accounting for 42% and 32% of the world’s capacity, respectively. Africa, with only 1 cardiothoracic surgeon per 4 million people, accounts for only 1% of the total global capacity^[21]^. This geographic maldistribution of the cardiothoracic surgical workforce serves as a clear indication that global equity in cardiothoracic surgery has not yet been achieved. Consequently, the Global Cardiothoracic Surgery Committee is prompted to advocate for immediate action, aiming to establish cardiothoracic surgery as a paramount global health priority.

Additionally, the progress of cardiothoracic surgery is hindered by the inadequate amount of research in this field. This issue is especially prevalent in LMICs located in Southeast Asia and Africa, where only a small percentage of research has been peer-reviewed. The reasons for this include obstacles such as language and financial barriers, a lack of regional journals, and insufficient support and capacity for research^[18]^. HICs produce 81.1% of the global CVD research output compared to 2.8% by LMICs^[22]^. It is worth noting that, unlike HICs, LMICs account for 59.5% and 57.1% of the global CVD morbidity and mortality rates, respectively^[22]^. However, despite being the primary source of morbidity and mortality from CVD globally, LMICs have the lowest research output in this field. Addressing this issue would involve implementing initiatives such as establishing partnerships between institutions in HICs and LMICs and providing opportunities for capacity-building. These measures aim to encourage researchers to conduct and publish research that contributes to the global cardiothoracic surgery literature in LMICs.

Conclusively, Cardiac surgery is a highly intricate field that requires the expertise of a multidisciplinary team and advanced infrastructure. Nevertheless, there remains a shortage of global capacity building in cardiothoracic surgery training for allied health professionals. This training is crucial in assisting cardiothoracic surgeons and reducing their workload. Also, there exists a significant discrepancy between cardiothoracic surgery programs in HICs and LMICs concerning the availability of surgical equipment and the adequacy of facilities. Moreover, this disparity is further exacerbated by the lack of literature dedicated to information management for cardiothoracic surgical care in LMICs. Nevertheless, the mentorship programs and collaborations known as the “North-South” and “South-South” initiatives are deemed crucial mechanisms for fulfilling the training requirements in the field of cardiac surgery^[15]^.

### The inherent benefits of strong cardiothoracic surgical capability for health systems

With its known ability to save lives and prevent/correct disabilities, surgical care has become increasingly important as part of the global health agenda in recent times. The significant interventional role of cardiothoracic surgeons in the treatment of cardiovascular diseases such as congenital heart disease (CHD), valvular and aortic diseases, rheumatic heart disease, and ischemic heart disease creates a unique niche for them within the field of medical practice^[23]^. Cardiothoracic surgeons comprise cardiac surgeons, general thoracic surgeons, and cardiovascular surgeons, who are specialized in surgery on the organs of the chest such as the heart, lungs, esophagus, and tissues of the bone that make up the chest wall^[24]^. In low- and middle-income countries, cardiac surgery is inaccessible to about six billion people and about 90% of the over one million babies born annually with congenital heart disease (CHD) also lack access to appropriate and timely treatment^[23]^. More importantly is that over 70% of these CHD patients require medical/surgical care within the first year of life, else they die or become severely disabled. In the United States, by 2030, the burden of heart failure and coronary heart diseases are projected to rise above the 2010 levels by 25% and 16.6%, respectively^[25]^.Therefore, this increasing rise in the burden of cardiovascular disease calls for a commensurate rise in the number of surgeons within the health system, since they offer special role no other practitioner offers—cardiothoracic surgeons are needed more than ever^[25]^.

### Global cardiac surgery initiative: purpose and vision

Driven by the fact that over 6 billion people worldwide lack access to safe cardiac surgical care and cardiovascular diseases accounting for over 17.5 million deaths per year and 80% of these mortalities occurring in low-income countries^[17]^, there was a need for an implementation of a movement which could advocate for global cardiac surgery. The Global Cardiac Surgery Initiative was founded in 2018^[26]^ with the purpose of advocacy, education, research, and sharing open access resources of global cardiac surgery to its members who are trainees or are already cardiac surgeons, especially from LMIC’s^[5]^ and thus through their purpose ensure that the next generation of surgeons are trained and equipped. However, some other societies have also been a big push to cardiothoracic surgery in a global and national health context through their numerous works (sessions on global cardiac surgery during annual meetings, engaging in establishment of cardiac surgery in LMIC’s) and aids in seeing cardiothoracic surgery included and becomes an integral part in global and national health systems, they include: Cardiac Surgery Intersociety Alliance (CSIA), which was put forward as a joint effort between most of the regional professional cardiac surgical societies globally, which include: American Association for Thoracic Surgery (AATS), European Association for Cardio- Thoracic Surgery (EACTS), Asian Society for Cardiovascular and Thoracic Surgery (ASCVTS), World Heart Foundation and the Society of Thoracic Surgeons (STS) with main objective to endorse cardiac surgery in LMIC’s by deriving metrics of performance and quality for the centers which have been endorsed, improving and standardizing clinical care to the best way possible^[5,27]^. Again, the African Association of Thoracic and Cardio-Vascular Surgeons (AATCVS) has over the years, helped in ensuring safe access to cardiac surgery especially within Africa and has improved significant cardiothoracic surgery in Africa through aids and annual meetings^[5]^.Also, the Canadian Cardiovascular Society stated in 1947, with the main objective to promote heart health for all by ensuring strong teams in a heart healthy Canada with key principles of: integrity, excellence and quality, community, health, evidence and diversity, equity and inclusion^[28,29]^ and has been of help in engaging cardiothoracic surgery within global health coverage. Finally, the Thoracic Surgery Foundation (TSF), an organization that seeks to support and promote the welfare of others and is largely supported by the STS, has increasingly supported cardiac surgery efforts globally. Through the Every Heartbeat Matters Award, in collaboration with Edwards Lifesciences, the TSF has supported multiple initiatives to train and care for healthcare professionals to manage valvular heart disease in variable-resource contexts particularly LMICs. TSF has provided multiple travel scholarships for trainees and surgeons to attend cardiothoracic surgery meetings and pursue mini- or full fellowships in centers abroad^[5]^.

## Discussion

### Way forward

Over the course of history, challenges have often been a driving force for advancement, and this is certainly true of cardiothoracic surgery. In the past, challenges often inspired individuals to pick up the mantle of cardiothoracic surgeons and strive towards innovative treatments leading to better quality of surgery and patient outcomes. In Africa today, as far as cardiothoracic surgery is concerned, surgeons face multifaceted challenges in their practice. The rapid poverty growth, bad governance, political instability, insufficient cardiothoracic surgeons, widespread corruption, and a lack of universal health coverage are among the impeding factors to the advancement of cardiothoracic surgery^[30]^. More so, with the doubling of cardiovascular disease prevalence between 1990 and 2019, and the improving socioeconomic status of underdeveloped countries, the utilization of cardiothoracic surgery for the care of patients is on the rise. The increasing aging demographics and the burden of chronic diseases requiring surgical intervention have also contributed to this recent rise. Therefore, it is becoming more important than ever to develop goals and objectives for the future that will be commensurate with such a rise in meeting the demands and care of patients^[25]^. Considering the unfavorable growth index in several countries, it may be considered practically impossible for the establishment of new and maintain existing infrastructures and equipment needed for the actualization of such surgery. Compared to the United States of America where there is open heart surgery for 150 000 inhabitants, Africa has one center for 50 million people. The death of human resources due to a shortage of skilled staff is a serious one that causes undervaluation of cardiovascular disease or prolongs the time of diagnosis. In combating this, more training programs should be developed especially in LMICs. More training centers for cardiothoracic surgery, clinical care, and research on open heart surgery should be promoted^[15]^. This should involve a team-based approach aimed at enhancing the capacity of members within the local context of their nations. These trainings should immerse participants into the local settings of their nation, allowing them to utilize the skills, resources, and facilities obtainable within the health system they function in, as opposed to training abroad. These trainings can benefit more from international collaboration whereby teams from high-income countries and LMICs engage one another, sharing and improving their knowledge and ideas about the delivery of cardiac surgery and training in advanced facilities where new skills and techniques can be acquired. A typical example of such a program is the Global Surgery and Implementation Science at the J.W. Lee Center for Global Medicine, Seoul National University, South Korea, which was established in 2012, to reduce health disparities through international cooperation and research with LMICs. The program focuses on two major activities which involve an in-country training in a given LMIC and an invitational training program in South Korea. The in-country training embeds participants in their local settings, which provides reality-driven and sustainable skill development while utilizing the workforce, resources, and facilities of the health system in which the providers function. On the other hand, the invitational program conducted bi-annually invites members from an LMIC to South Korea to observe and assist with surgeries at the Seoul National University Hospital. By so doing, team members from LMICs learn new techniques for performing complex surgeries and keep up-to-date with the latest technologies. Several countries such as China, Nepal, Vietnam, Ivory Coast, Uzbekistan, Laos Mongolia, and Ethiopia have benefitted from this training program^23^. Also, in countries, especially the LMICs, which do not have cardiac surgery listed as a top priority in any official documents, attention is more often placed on communicable diseases and maternal and child health at the expense of non-communicable disease prevention efforts. However, with the recent rise in non-communicable diseases, and CVDs accounting for a significant portion of them, structural conditions such as congenital heart disease and rheumatic heart disease have contributed to a high yield of high morbidity and mortality. These countries should, therefore, adopt the global surgery initiatives into their ministries of health and the needed resources should be allocated to build infrastructural facilities which will encourage surgeons to work and stay in the system and effectively implement surgical procedures. By so doing, more cardiothoracic surgeons will become actively involved in the national health dialogue, helping to meet the prevailing challenges^[14]^.

## Conclusion

The chronic shortage of cardiothoracic surgical workforce in LICs is no longer a secret. Over the years, many resources have been mobilized to curb this deficit but most of them, if not all these initiatives did not solved the problem effectively; they rather attenuated the situation temporarily. With the predicted increase in the prevalence of patients in need of cardiac surgical care, it is imperative that LICs put more effort in reinforcing the cardiac surgical workforce. Nonetheless, the need for LICs to invest in building their respective cardiac surgical workforces does not exclude the necessity for collaborations with HICs who usually have a more developed cardiac surgical workforce. The wide gap between high and low-income countries in terms of the prevalence of heart diseases and the cardiac surgical workforce poses an immense constraint to Global cardiac surgery. These disparities could be attributed to the insufficient advocacy from various cardiac surgery-related organizations and associations, as well as the paucity of research interventions to concretize the current state of cardiac surgery in LICs.

Changing the status quo of global cardiac surgery will require concerted and collaborative efforts between governmental and non-governmental institutions, as well as other related bodies, to address the numerous gaps in global cardiac surgery.

### Limitation

The inclusion criteria focus on open-access and peer-reviewed journals, which may exclude some valuable unpublished or non-English-language research, thereby limiting our scope of data curation.

## Data Availability

This article did not report any results derived from research data.

## Research registration unique identifying number (UIN)

Not required.
